# Abstract of the Proceedings of the Buffalo Medical Association

**Published:** 1856-06

**Authors:** Thos. F. Rochester


ART. V.—*Abstract of the Proceedings of the Buffalo Medical Association.*

Present—Drs. Flint, White, Strong, Howell, Lemon, Wilcox, Nichols. and Rochester.

The society was called to order by Dr. Flint, the vice-President; and Dr Rochester was appointed secretary pro. tem.

The reading of the minutes of the preceding meeting was dispensed with.

Individual reports on prevailing diseases being in order, all present stated that diarrhœa was unusually prevalent. Dr. Wilcox said that it seemed, in many instances, to assume the form of colitis, being associated with colicky pain, and tenderness along the track of the large intestine. He had found treatment with anodynes, astringents, and tonics, effectual, particularly the oxide of bismuth.

*Dr. Strong* reported a case of tuberculosis of long standing, in which, however, the abdominal symptoms, such as diarrhœa, tympanitis, et. cet. preponderated over the thoracic. The patient has now fistula in ano, and Dr. Strong would be pleased to hear the opinion of the members present, as to the propriety of a surgical operation for the relief of the fistula; he conceived that the commonly received opinion, that the healing of the fistula was apt to be followed by an increase of the tubercular disease in the lungs was erroneous—that inasmuch as the fistula tended to debilitate the patient—it would thereby promote the development of pulmonary tuberculosis.

*Dr. Wilcox* had had a patient with tuberculosis pulmonalis, who also had two fistulæ, upon which he had operated with setons with success; the general health of the patient had improved.

*Dr. Rochester* had seen three instances of operation for fistula in ano, in tubercular subjects; two of them died in less than two years of pulmonary tuberculosis, and he was under the impression that the third had also died within that period; he thought an operation extremely hazardous in such cases, and likely to abridge the life of the patient.

*Dr. Flint* cited an instance of fatal pulmonary tuberculosis following the healing of a fistula in ano; he thought, however, that the inquiry of Dr. Strong was one of much interest, and suggested that a committee be appointed to report upon the subject.

After a few remarks by Drs. Strong, White, Baker, and Rochester, it was moved by Dr. Baker, and seconded by Dr. Rochester, that Dr. Strong be appointed to prepare and to present to the society, at his convenience, a paper on "Fistula in Ano in its relations to Pulmonary Tuberculosis." Carried.

*Dr. White* then reported a severe case of uterine hæmorrhage, following abortion at three months, to which he had been called by Dr. Wm. Newell, who had employed the usual remedies, including the tampon, without avail. The embryo had been expelled with its membranes unbroken and enveloping it. The patient was healthy, aged 27, and this was her eleventh (?) conception. Dr. White at once made a vaginal examination; he found the os uteri patulous, and detected just within it a large fleshy mass; this he removed, and the uterus at once contracted and the hæmorrhage ceased. The tumor, on examination, proved to be a blighted ovum twin to a perfect embryo. This Dr. W. thought very unusual. There was a question of interest in the speaker's mind, could this false growth have preceded the true conception? it was a very different thing from superfœtation, which of course he did not believe in. In reply to an inquiry from the vice-president, Dr. W. said that a blighted ovum, or false conception, was a mass, in which the embryo had perished, of membranes and placenta which continued to augment in size for various periods; that no definite idea, however, of time could be determined by the size of the mass; blighted ova were probably the cause of more abortions, so called, than mere indiscretions on the part of the female.

Dr. White also reported a case of much interest, of which he became at first cognizant almost accidentally. He was called to see a child in a family that he regularly attended, and as he concluded his visit, the mother of the child remarked that she was not herself very well. On inquiry he found that she complained of lassitude, faintness, diminished appetite, and of constant thirst; that her skin was dry, and that she had been losing flesh during the winter; the tongue was raw and red. The aspect of the tongue, the emaciation, the thirst, and the aridity of the skin, caused Dr. White at once to suspect diabetes mellitus. The patient replied, to interrogatories, that she was not aware of passing an unusual amount of urine. The quantity for twenty-four hours, however, on measurement, proved to be seven quarts, and with a sp. gr. of 1.037. A portion being subjected to Moore's and Trommer's tests, the characteristic oxide of copper reäction of saccharine urine was established.

The patient was placed upon an exclusively animal diet, and was directed to employ warm alkaline baths every other day, and to wear thick flannel next the surface. This plan was continued for three weeks without any improvement. On one occasion after the administration of a full dose of Paregoric, for the relief of abdominal pain, the quantity of urine was sensibly lessened. It was now determined to change the treatment, Dr. Rochester, who was visiting the patient with him, concurring. The alkaline baths were directed to be continued, and Trousseau's alkaline method was resorted to, viz., the daily administration of from ʒij to ʒviij of the bi carb. of soda largely diluted with water, the patient being allowed to eat freely of green vegetables; this last measure seemed necessary, from the patient's absoolute loathing of animal food. At the end of one month of this treatment, there was no improvement as to the quantity or quality of the urine, the sp. gr. being increased to 1.042; the thirst, however, was less urgent, the bowels were less confined, and the skin was less dry. The soda was suspended and 20th of a grain of strychnine, three times a day, was ordered, as recommended by Fricke, of Baltimore. This last was only employed for a few days on account of its unpleasant effects upon the nervous system. No improvement followed its use. The patient is now traveling to try change of air and a warm climate.

*Dr. Holmes* stated that he had seen patients with diabetes treated successfully in Valleix's wards, with animal food and pure *gluten*.

There being no further business, on motion, adjourned.

THOS. F. ROCHESTER, Sec'y, pro. tem.

